# MIA3 promotes the degradation of GSH (glutathione) by binding to CHAC1, thereby promoting the progression of hepatocellular carcinoma

**DOI:** 10.1007/s11010-023-04850-9

**Published:** 2023-11-10

**Authors:** Zhou Wanbiao, Man Jing, Zuo Shi, Chen Tengxiang, Zhao Xueke, Li Haiyang

**Affiliations:** 1 Department of Hepatobiliary Surgery, The Affiliated Hospital of Guizhou Medical University, 28 Guiyi Street, Guiyang, Guizhou People’s Republic of China; 2 School of Clinical Medicine, Guizhou Medical University, Guiyang, Guizhou People’s Republic of China; 3 Department of Infectious Diseases, The Affiliated Hospital of Guizhou Medical University, Guiyang, Guizhou People’s Republic of China

**Keywords:** MIA3 (melanoma inhibiting active protein 3), TANGO1 (Golgi transporter component protein), CHAC1 (glutathione-specific γ-glutamyl cyclotransferase 1), GSH (glutathione), Hepatocellular carcinoma, Prognosis

## Abstract

MIA3 (melanoma inhibitory active protein 3)/TANGO1 (Golgi transporter component protein) plays an important role in the initiation, development, and metabolism of cancer. We aimed to explore the role and underlying molecular mechanisms of MIA3/TANGO1 in the growth and migration of hepatoma cells. According to the analysis of The Cancer Genome Atlas (TCGA) database, MIA3 is expressed at higher levels in hepatocellular carcinoma (HCC) tissues than in normal tissues. Real-time quantitative polymerase chain reaction (qRT-PCR), immunohistochemistry, and western blotting were used to detect mRNA and protein expression in HCC tissues and cells. The in vitro function of MIA3 in HCC cells was evaluated using Cell Counting Kit-8 (CCK-8), colony formation, cell migration and invasion, and flow cytometry assays. Hep-G2 cells with MIA3 overexpression were subjected to RNA-seq, and the downstream target gene CHAC1 (glutathione-specific γ-glutamyl cyclotransferase 1) was selected according to the results of the volcano map of gene enrichment. The relationship between MIA3 and CHAC1 was revealed by coimmunoprecipitation and confocal microscopy. MIA3 expression was upregulated in HCC organizations and HCC samples in the TCGA dataset. Knocking out MIA3 inhibited the proliferation, migration, and invasion of Hep-G2 cells and promoted the apoptosis of Hep-G2 cells. Overexpression of MIA3 in Huh7 cells promoted the proliferation, migration, and invasion and suppressed the apoptosis of Huh7 cells. Overexpression of MIA3 promoted the expression of CHAC1 and the degradation of glutathione (GSH), thereby promoting the growth and metastasis of HCC cells. Knocking out MIA3 inhibited the expression of CHAC1 and slowed the degradation of glutathione, thereby inhibiting the growth and metastasis of HCC cells. MIA3 further promotes the growth, metastasis, and invasion of hepatoma cells by binding to the CHAC1 protein and promoting GSH degradation.

## Introduction

Liver cancer is one of the most common malignant tumours, and hepatocellular carcinoma is the most common type. In 2020, liver cancer ranked sixth among cancers in terms of incidence and third in terms of mortality rate; it accounted for approximately 906,000 new cases and 830,000 deaths. Early-onset liver cancer has an insidious onset and a poor prognosis; it is difficult to diagnose, and treatments are limited, which seriously threatens human physical and psychological health [1]. Therefore, there is an urgent need to study the underlying molecular mechanisms of liver cancer to provide new diagnostic biomarkers and treatment targets for liver cancer.

Melanoma inhibition active protein 3 (MIA3), also known as TANGO1, is a member of the MIA gene family and is an intact membrane protein localized to the endoplasmic reticulum (ER) outlet. Existing studies have shown that MIA3 promotes collagen VII output [2]. In oral cancer, the expression of the MIA3 gene promotes the proliferation and migration ability of oral squamous cell carcinoma cells and inhibits apoptosis of cancer cells [3]. In colorectal cancer, overexpression of the MIA3 gene inhibits the proliferation and migration capacity of colorectal cancer cells [4, 5]. In coronary artery disease, expression of the MIA3 gene accelerates disease progression [6]. In mouse experiments, MIA3 deletion led to defects in collagen transport and secretion, which further led to severe impairment of mouse embryonic chondrocyte maturation and bone calcification [7]. However, the mechanism of MIA3 in HCC disease is unknown.

Recent studies have shown that glutathione-specific γ-glutamyl cyclotransferase 1 (CHAC1) is a member of the γ-glutamyl cyclotransferase family involved in the γ-glutamyl cycle [8, 9]. CHAC1 degrades glutathione (GSH), can catalyse the cleavage of GSH to 5-oxo proline and the dipeptide cysteinylglycine and is one of the only known cytosolic enzymes that can degrade GSH [8, 10]. Overexpression of CHAC1 leads to GSH depletion and alteration of cellular redox balance [8, 9]. CHAC1 has also been identified as a component of the unfolded protein stress signalling pathway in the endoplasmic reticulum (ER) [11, 12]. Elevated levels of CHAC1 mRNA in gastric, breast, and ovarian cancers are also associated with a poor prognosis [13–18].

We previously found that MIA3 protein levels are significantly elevated in both HCC patients and HCC cell lines compared to corresponding controls. MIA3 upregulation is related to clinical progression and a poor prognosis in HCC patients. In this study, in vitro and in vivo experiments showed that MIA3 further promotes the proliferation, migration, and invasion of HCC cells by binding to CHAC1 and promoting the degradation of GSH. Therefore, the MIA3/CHAC1/GSH axis may be a new target for the diagnosis and treatment of hepatocellular carcinoma.

## Materials and methods

### Bioinformatics analysis

RNA sequencing data for a total of 371 LIHC samples and 50 normal samples and corresponding clinical information were downloaded from the TCGA database and processed using the biological conductor software package in the R language statistical environment.

### Cell culture

A normal liver cell line (LO2) and HCC cell lines (HCCLM3, Hep-G2, Huh7, and Hep-3B) were purchased from the Chinese Academy of Sciences Cell Bank (Shanghai, China); the cells were cultured in DMEM (GibcoBRL, Rockville, MD, USA) with 10% foetal bovine serum (04-001-1A, Biological Industries) and incubated at 37 °C and 5% carbon dioxide.

### Quantitative real-time reverse transcription polymerase chain reaction

Total RNA was isolated from HCC cell lines using the TRIzol kit (Invitrogen). The cDNA synthesis kit (YEASEN, China, 11141ES) was used to synthesize cDNA, cDNA was used as a template, and specific primers were used for amplification (GAPDH gene as an internal gene control). A qRT-PCR kit (YEASEN, China, 11184ES) was used for qRT-PCR. The 2 ^(−ΔΔCt)^ method was used to calculate the relative gene expression. GAPDH was used as an internal reference for normalization.

The primer sequences were MIA3 forward: 5′-TGACCGCAACTCACTACAA-3′; reverse: 5′-AAAGCCATC TCCTTCTGCT-3′;

GAPDH, forward: 5′-GATCATCAGCAATGCCTC-3′;

reverse: 5′-GTCCTTCCACGATACCAA-3′.

CHAC1, forward: 5′-GACGCTCCTTGAAGATCATGAG-3′; reverse: 5′-CAGCAAGTATTCAAGGTTGTGG-3′.

TGM2,forward: 5′-AATCCAGAAATCAAGATCCGGA-3′;

reverse: 5′-CAGGTCCATTCTCACCTTAACT-3′.

CEBPB, forward: 5′-TTATAAACCTCCCGCTCGGC-3′;

reverse: 5′-TTCCATGGGTCTAAAGGCGG-3′.

### Immunohistochemistry

A total of 92 pairs of primary liver cancer tissues were analysed using tissue chips from Shanghai Xinchao Biotechnology Co., Ltd, and related clinical and prognosis data were available. Immunohistochemistry (IHC) staining was used to detect the expression of proliferating nuclear antigen (PCNA) and Ki67 in tissues. Tumour xenograft tissue was deparaffinized, hydrated, and subjected to antigen retrieval; 5% BSA was added to block the nonspecific binding site for 20 min. Tissue sections were incubated overnight with primary antibody in a 4 °C freezer, and then secondary antibodies were added and incubated for 60 min at room temperature. After 5 min of incubation with chromogenic diaminobenzidine at room temperature, the degree of staining of the antigen was observed under a microscope. Antibodies against MIA3 (1:100), PCNA (1:200; protein proteins), and Ki67 (1:100; Cell Signaling Technology) were used; haematoxylin stain was also applied, and hydrochloric-alcohol was used to induce differentiation.

### Lentivirus infection

MIA3 lentiviral particles were constructed by Biomedicine company (Chongqing, China). Hep-G2 and Huh7 cells were infected with lentiviral vectors (including experimental lentiviral vectors and empty negative controls). HCC cells were seeded in 6-well plates, and when the confluency level reached 40–50%, 1 ml of lentiviral (MOI: 10–40) containing fresh medium was added to each well. Hep-G2 and Huh7 cells were infected and divided into experimental and negative control groups. After 72 h, the fluorescence expression efficiency of HCC cells was detected with fluorescence microscopy, followed by screening for stably transfected cells with 1–10 μg/ml puromycin. The MIA3 overexpression and knockout efficiency were measured by qRT-PCR, and Western blotting was performed for further verification.

### SiRNA transfection

MIA3 siRNAs were designed and synthesized by GeneChem (Shanghai, China). To inhibit the expression of the CHAC1 gene, we transfected 10 nM siRNA into HCC cells using lipofectamine 3000 reagent (Invitrogen) following the manufacturer's procedure.

### Colony formation experiment

Cells were seeded in 6-well plates at a rate of 200–500 cells/well, and after 14 days of incubation with 10% foetal bovine serum medium, the cells were washed twice with PBS solution and stained with haematoxylin solution. The colonies were counted under the microscope.

### Cell proliferation assay

CCK-8 (Beyotime, China, C0037) and EdU (BeyoClick™ EdU-594 Cell Proliferation Kit, Beyotime, China, C0078S) assays were used to perform cell proliferation analysis according to the manufacturer's instructions. EdU staining was observed with a Lycra laser scanning microscope.

### Cell migration and invasion assays

Wound healing experiments and transport assays were used to assess cell migration and invasion capacity. Cells were seeded into 6-well plates, and when the cells reached 100% confluence, a scratch was made across each well with a 200 μl pipette tip. Photos were taken and recorded at 0 h. The cells were cultured in serum-free medium for 48 or 72 h, and the wound was photographed to assess wound closure. For transwell experiments, cells were seeded at a density of 5 × 10^4^ cells/well in a 24-well polycarbonate transport filter (8 μm pore size, Corning, USA), precoated (invasion assay) or not precoated (migration assay) with 30 μL matrix and 20% foetal bovine serum (500 μl) in the lower chamber. After 24 or 48 h of incubation, cells were fixed with 4% paraformaldehyde and stained with 0.1% crystal violet. The cells in the top chamber were wiped away with a cotton swab, and the cells that migrated through to membrane were photographed with an inverted microscope.

### Apoptosis and cell cycle analysis

Apoptosis and apoptosis assay kits (Beyotime, China, C1052) were used to detect apoptosis. Cell cycle analysis with propidium iodide (PI) staining. NAVIOS flow cytometry (Beckman Navios) was used to detect apoptosis and cycle distribution.

### Determination of glutathione levels

The glutathione content was detected by a reducing glutathione (GSH) content detection kit (Solarbio, China, BC1175). According to the reagent vendor's protocol, 200 μL of the solution was added to 5 × 10^6^ cells, mixed with shaking, and allowed to stand for 2 min. The glutathione content was corrected according to the protein concentration per milligram, and the optical density (OD) was measured at 412 nm.

### Animal research

The animal research was approved by the Animal Ethics Committee of the Affiliated Hospital of Guizhou Medical University (approval number 2201480). A total of 1 × 10^7^ MIA3-overexpressing Huh7 cells in the logarithmic growth phase and corresponding negative control (NC) cells were resuspended in 150 µl with autoclaved PBS and injected into the subcutaneous tissue of female nude mice (BALB/C, females 3–4 weeks old) (5 in each group, 2 groups). Tumour volume was recorded every 7 days. On day 21, the tumour tissue was removed and weighed. Subsequently, HE and IHC experiments were carried out. All animal studies were conducted in accordance with the principles and procedures outlined in the Guidelines for Animal Care and Use of Guizhou Medical University.

### Western blot analysis

The Western blot detection methods were described in a previous article [19]. Primary antibodies included anti-MIA3 (1:1000, Proteintech, China 17,481–1-AP), anti-E-cadherin (1:5,000, Proteintech, China, 20,874–1-AP), N-cadherin (1:6,000, Proteintech, China, 22,018–1- AP), anti-Vimentin (1:5,000, Abcam, USA, ab92547), anti-cyclin D1 (1:1,500, Proteintech, China, 26,939–1-AP), anti-cleaved caspase-3 (1:500, Abcam, USA, AB32042), and anti-CHAC1 (1:1000, Proteintech, China, 15,207–1-AP).

### Coimmunoprecipitation (Co-IP) analysis

Cells were collected, lysed with RIPA buffer at 4 °C for 30 min, and centrifuged at 12,000 rpm/min for 10 min; the supernatant was removed, and then the sample was incubated with immunoglobulin beads for 2 h at 4 °C and centrifuged at 3,000 rpm for 10 min. The supernatant (20 μl) was stored as “Input”, and anti-IgG antibodies (Proteintech, Wuhan, China), anti-MIA3 antibodies (Proteintech, Wuhan, China, 17,481-1-AP), and anti-CHAC1 antibodies (Proteintech, Wuhan, China) were added to the remaining cell lysate. The cell lysate containing the antibody was gently swirled overnight at 4 °C. Protein A/G beads were added to the cell lysate and spun down at 4 °C for 4 h to obtain the beads. The beads were washed, eluted in sample buffer, and boiled at 100 °C. Western blot analysis of immune complexes was then performed.

### Confocal microscopy

Huh7 and Hep-G2 cells were cultured overnight (2 × 10^5^/well) and then fixed with 4% paraformaldehyde and infiltrated with 0.5% Triton X-100 for 20 min at room temperature. HCC cells were incubated overnight with MIA3 (Proteintech) antibody and CHAC1 (Proteintech) antibody at 4 °C. The cells were stained with secondary antibodies for 2 h in the dark at 37 °C, and the nuclei of HCC cells were stained with DAPI. Images were taken using a laser scanning confocal microscope.

### Transcriptomic data analytics

Hep-G2 cells transfected with MIA3 overexpression vector or with an empty viral vector as the control were constructed and divided into an experimental group and a blank control group, and the transfection efficiency was measured by qRT-PCR. Three biological replicates of 1 × 10^7^ cells per group were obtained from the experimental group and the blank control group, and transcriptome sequencing was performed using the Illumina high-throughput sequencing platform. After obtaining sequence expression data for each sample, differentially expressed genes in each control group were analysed using edgeR. The p value was corrected for multiple hypothesis tests using the BH method. We selected fold change ≥ 2 (i.e. the absolute value of log2FC ≥ 1) and q value < 0.05 (q value is the corrected value of p value) as the criteria for screening differentially expressed genes. GO annotation and KEGG enrichment analysis were performed on differentially expressed genes to determine their biological roles.

### Statistical analysis

Statistical analysis was performed using the SPSS 22.0 statistical software package (SPSS, Chicago, IL, USA). Two groups were compared using Student's t test, and more than two groups were compared using one-way ANOVA. GraphPad Prism 8 (GraphPad software, USA) was used for statistical analysis and graph generation. Survival analysis was performed using the Kaplan‒Meier method. All experiments were performed independently three times. A *p* value < 0.05 indicates a significant difference (*p < 0.05, **p < 0.01, ***p < 0.001).

## Results

### Overexpression of MIA3 is correlated to hepatoma cells

**Fig. 1:**
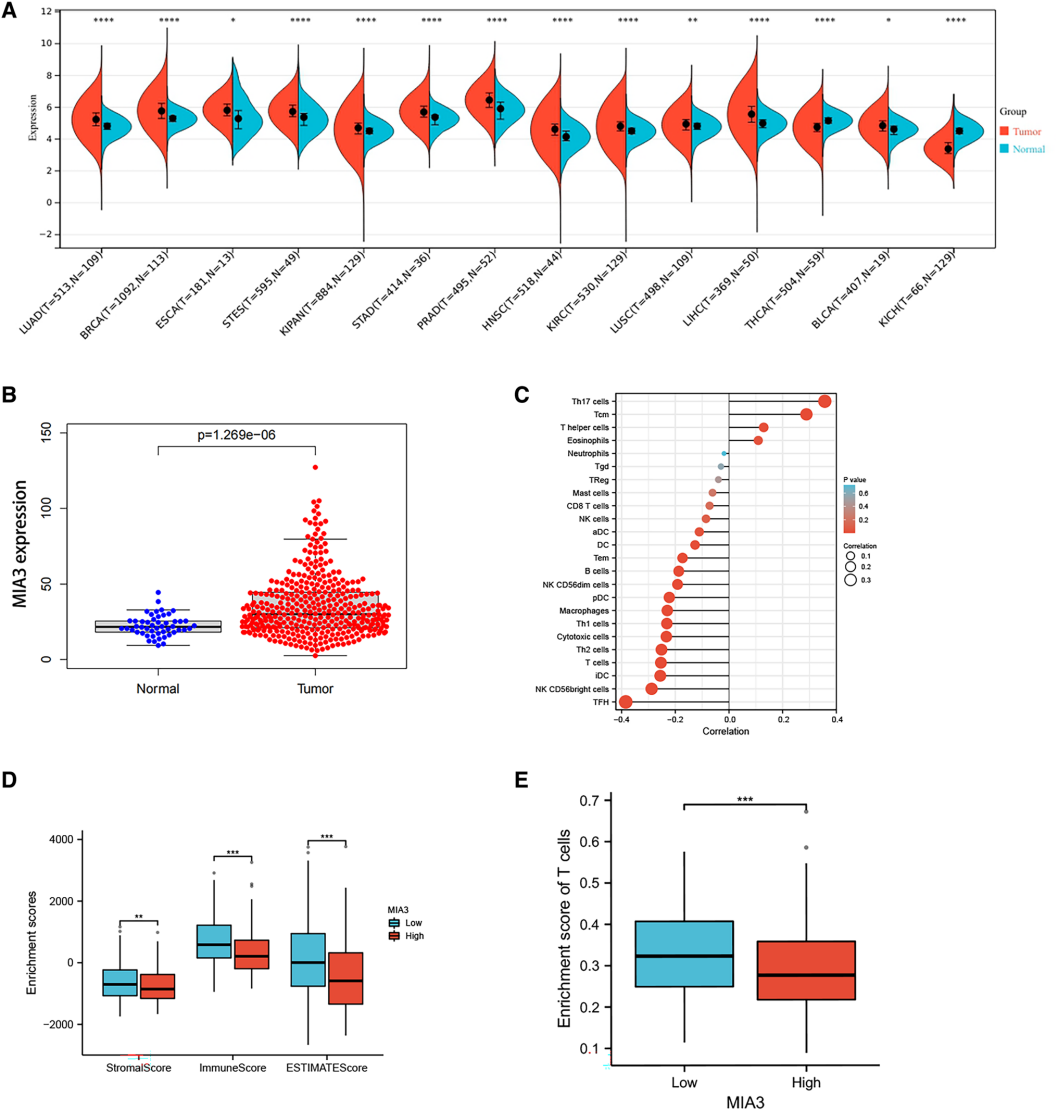
Differential expression of MIA3 in tumours. **A** Pancancer analysis based on The Cancer Genome Atlas (TCGA) database. **B** Expression of MIA3 in HCC samples based on the TCGA database. **C** MIA3 and immune cell infiltration analysis based on TCGA database. **D** Differences in immune matrix scores of MIA3 based on the TCGA database. **E** Differences between high and low expression groups of MIA3 in T cells based on the TCGA database. (*p < 0.05, **p < 0.01, ***p < 0.001)

We compared MIA3 expression in different types of tumour samples and corresponding normal samples using liver cancer sequencing data from the TCGA database. We found that MIA3 expression was significantly altered in lung adenocarcinoma (LUAD), breast invasive carcinoma (BRCA), oesophageal cancer (ESCA), gastric and oesophageal cancer (STES), mixed kidney cancer (KIPAN), gastric cancer (STAD), prostate cancer (PRAD), head and neck squamous cell carcinoma (HNSC), clear cell carcinoma of the kidney (KIRC), lung squamous cell carcinoma (LUSC), and hepatocellular carcinoma (LIHC) samples versus control samples. MIA3 was upregulated in bladder urothelial carcinoma (BLCA) and downregulated in thyroid cancer (THCA) and renal chromophobic cell carcinoma (KICH) (Fig. 1A, B). The results of immune infiltration analysis showed that high expression of MIA3 was correlated with decreased immune cell infiltration (Fig. 1C), and analysis of the immune matrix score showed that the StromalScore, ImmuneScore, and ESTIMATEScore were higher in the low MIA3 expression group than in the high MIA3 expression group (Fig. 1D). T cells were also more enriched in the low MIA3 expression group vs. the high MIA3 expression group (Fig. 1E).

### Upregulation of MIA3 in liver cancer is associated with a poor prognosis

**Fig. 2:**
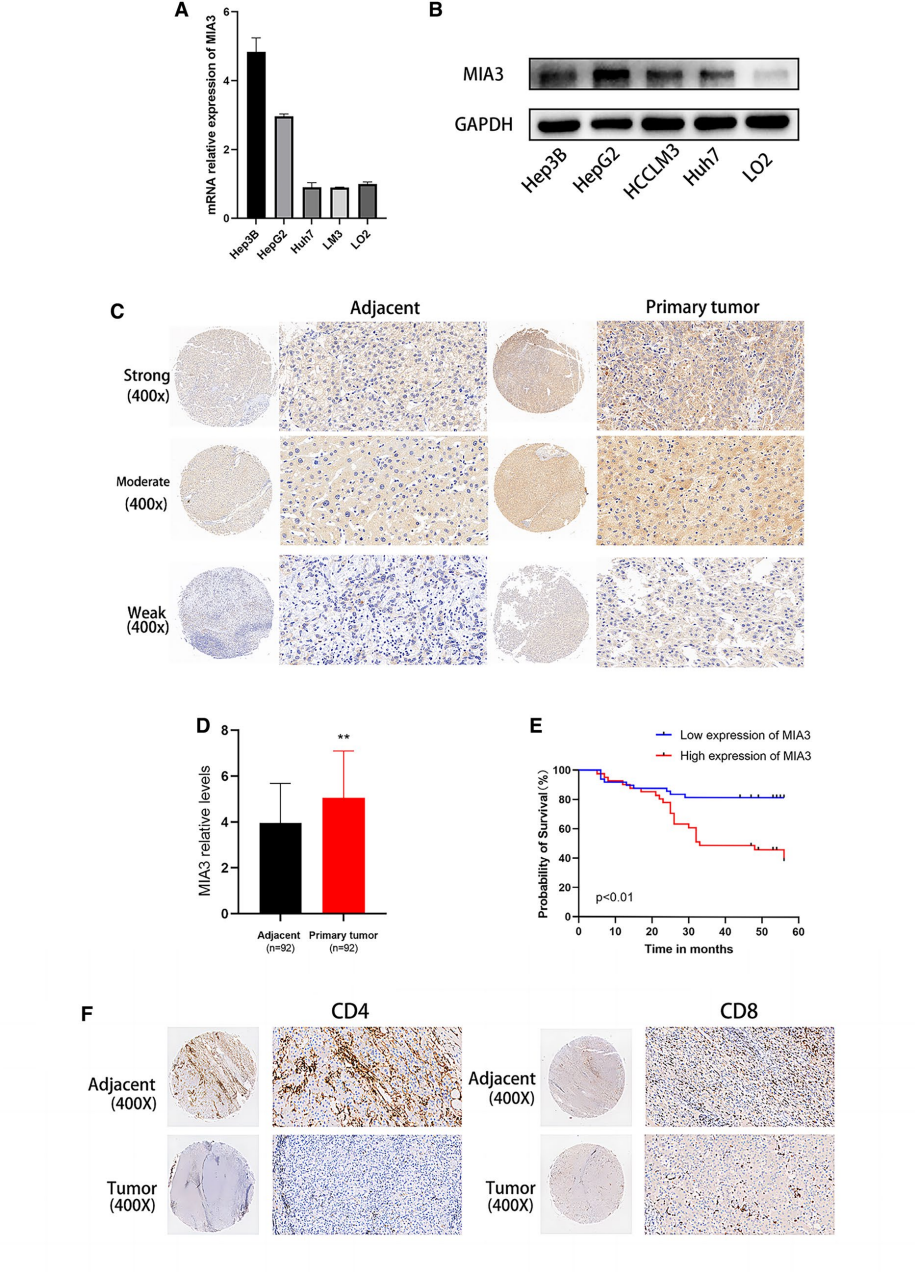
MIA3 expression in HCC samples. **A** Detection of mRNA expression levels of MIA3 in HCC cell lines by qRT-PCR. **B** Detection of protein expression levels of MIA3 in HCC cell lines by western blotting. **C** Immunohistochemistry detected the expression of MIA3 in HCC tissue chips. **D** Immunohistochemical scores of 92 cases of HCC and adjacent cancerous tissues in tissue chips. **E** Kaplan‒Meier survival analysis was used to evaluate the effect of MIA3 on 5-year survival. **F** Immunohistochemistry detected the expression of CD4 and CD8 in immune T cells in HCC tissue chips. (*p < 0.05, **p < 0.01, ***p < 0.001)

We confirmed that MIA3 is upregulated at the mRNA and protein levels in tumour cells by real-time quantitative PCR (qRT-PCR) and Western blotting of human normal and hepatoma cell lines (Fig. 2A, B). Immunohistochemical analysis of MIA3 in a tissue microarray (TMA) containing 92 pairs of HCC cancer tissue and adjacent nontumor tissue samples was performed by scoring cell staining (Fig. 2C, D), and survival analysis based on these results showed that overexpression of MIA3 is an unfavourable prognostic factor in HCC related to decreased overall survival (Fig. 2E). Immunohistochemical analysis of CD4 and CD8 T cells in a tissue microarray (TMA) containing 92 pairs of HCC cancer tissue and adjacent nontumor tissue samples was performed (Fig. 2F). We then evaluated the clinical significance of MIA3 expression (Table 1). The survival-related features identified by univariate Cox regression analysis were clinical stage (*p* < 0.001), pathologic grade (*p* = 0.001), tumour capsule status (*p* = 0.002), vascular invasion status (*p* < 0.001), and MIA3 expression (*p* < 0.001). However, multivariate Cox regression analysis showed that vascular invasion (p = 0.022) was better a predictor of poor survival than high MIA3 expression (Table 2).

**Table 1:** Clinical significance of MIA3 expression

Characteristics	*n*	MIA3 expression		
		High	Low	*P*
*Gender*				
Male	82	22 (26.8%)	60 (73.2%)	0.934
Female	10	2 (20%)	8 (80%)	
*Age (year)*				
≤50	40	7 (17.5)	33 (82.5%)	0.067
＞50	52	18 (34.6%)	34 (65.4%)	
*Clinical stage*				
I	59	15 (25.4%)	44 (74.6%)	0.846
II–III	33	9 (27.3%)	24 (72.7%)	
*Histological grade*				
I–II	44	7 (15.9%)	37 (84.1%)	0.033
III	48	17 (35.4%)	31 (64.6%)	
*Vital states*				
Alive	59	46 (78%)	13 (22%)	0.236
Die	33	22 (66.7%)	11 (33.3%)	
*Tumor scale*				
≤ 5cm	64	19 (29.7%)	45 (70.3%)	0.234
＞ 5cm	28	5 (17.9%)	23 (82.1%)	
*Recurrence*				
No	42	11 (26.2%)	31 (73.8%)	0.983
Yes	50	13 (26.0%)	37 (74.0%)	
*HBsAg*				
+	72	18 (25.0%)	54 (75.0%)	0.652
–	20	6 (30.0%)	14 (70.0%)	
*Vascular invasion*				
+	33	9 (27.3%)	24 (72.7%)	0.987
–	59	16 (27.1%)	43 (72.9%)	
*AFP*				
≤ 200	55	14 (25.5%)	41 (74.5%)	0.902
＞ 200	37	9 (24.3%)	28 (75.7%)	
*Involucrum*				
Complete	50	12 (24.0%)	38 (76.0%)	0.619
Incomplete	42	12 (28.6%)	30 (71.4%)

**Table 2:** Survival than high MIA3 expression

Parameter	Univariate analysis			Multivariate analysis		
	*P*	HR	95%CI	*P*	HR	95%CI
Gender	0.363	1.943	0.465–8.120			
Age (year)	0.901	1.002	0.970–1.035			
Histological grade	0.001	0.240	0.107–0.539	0.047	0.417	0.176–0.990
Tumor scale	0.090	1.076	0.989–1.171			
Recurrence	0.244	1.536	0.746–3.164			
Involucrum	0.002	3.206	1.542–6.665	0.038	2.210	1.046–4.669
Vascular invasion	< 0.001	7.354	3.301–16.383	0.022	3.205	1.186–8.661
MIA3	< 0.001	5.120	2.552–10.270	0.043	2.201	1.026–4.720
Clinical Stage	< 0.001	4.078	2.530–6.572	0.047	1.834	1.007–3.338
HBsAg	0.953	0.975	0.418–2.276			
AFP	0.799	1.000	1.000–1.000			

### Overexpression of MIA3 promotes the proliferation and migration of HCC cells

**Fig. 3:**
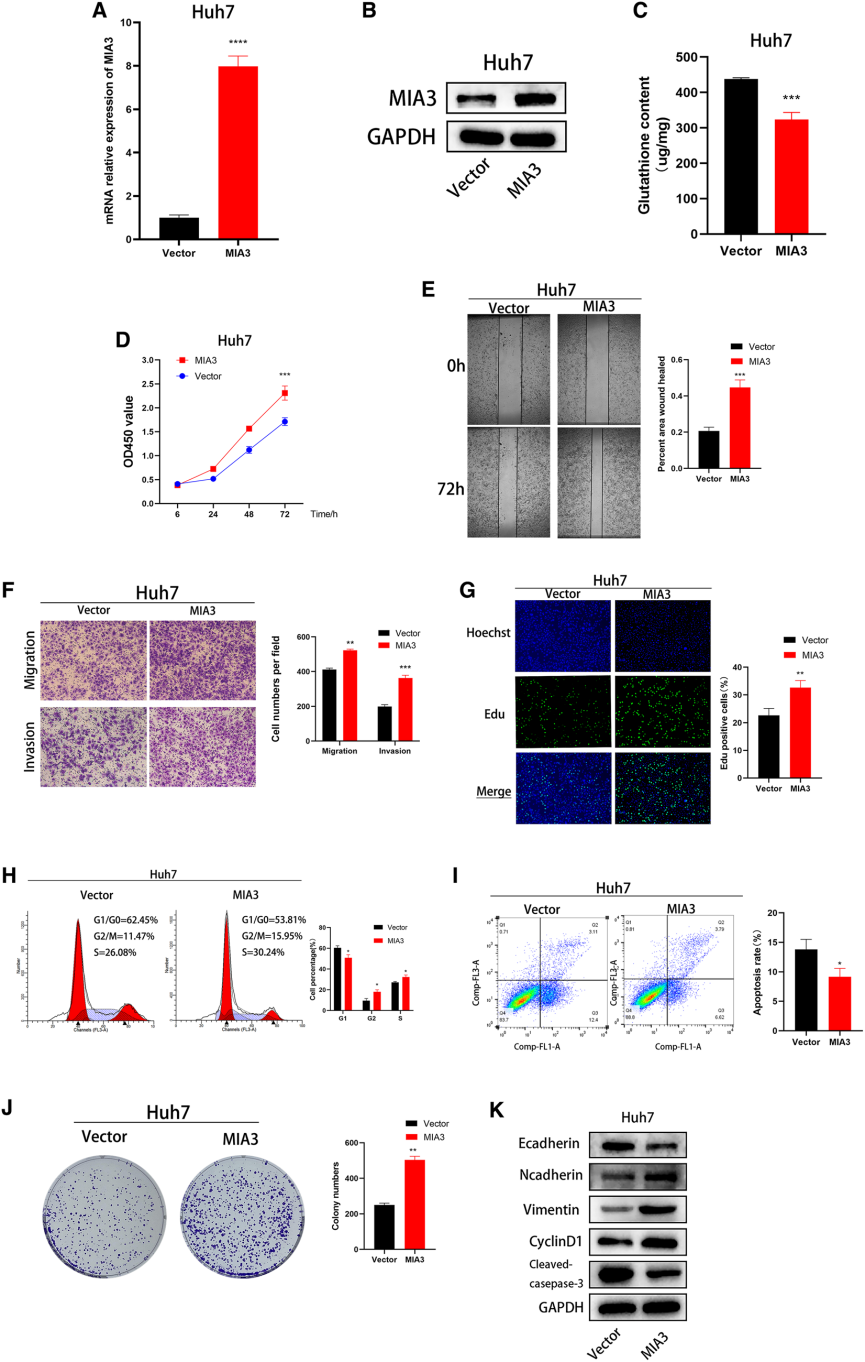
Upregulation of MIA3 promotes HCC cell growth. **A**, **B** After MIA3 lentivirus transfection into Huh7 cells, MIA3 gene expression at the mRNA and protein levels was measured by qRT-PCR and Western blot experiments. **C** Detection of glutathione expression in HCC cells after upregulation of MIA3. **D** CCK-8 experiments detected the proliferation function of HCC cells after upregulating MIA3. **E** Detection of the migration capacity of HCC cells with MIA3 overexpression by wound healing assay. **F** Detection of the migration and invasion ability of HCC cells with MIA3 upregulation via transwell assays. **G** EdU experiments detected the proliferation of HCC cells after MIA3 overexpression. **H**, **I** Flow cytometry analysis of the cell cycle distribution and apoptosis ratio of Huh7 cells. **J** Colony formation experiment to detect the proliferation of HCC cells after upregulation of MIA3. **K** Western blotting was used to detect proteins related to apoptosis, the cell cycle, and EMT in Huh7 cells. An unpaired two-tailed Student’s *t* test was used. All data are shown as the mean ± SD; (*p < 0.05, **p < 0.01, ****p < 0.001 for 3 independent experiments)

To explore the biological function of MIA3 in HCC cells, we established a stable model of MIA3 overexpression by lentiviral transduction of Huh7 cells, which have low basal expression of MIA3. The qRT-PCR and Western blotting results showed that MIA3 was effectively overexpressed in Huh7 cells (Fig. 3A, B). In Huh7 cells, the glutathione expression level in the MIA3 overexpression group was lower than that in the MIA3 vector control group (Fig. 3C). CCK-8, colony formation and EdU experiments showed that the overexpression of MIA3 promoted the proliferation of Huh7 cells (Fig. 3D, G, J). Cell migration and invasion experiments showed that the overexpression of MIA3 promoted the migration and aggressiveness of HCC cells (Fig. 3E, F). Cell cycle distribution analysis also showed that overexpression of MIA3 reduced the number of cells in the G1/G0 phase and increased the number of cells in the G2/M and S phases (Fig. 3H). Apoptosis experiments showed that overexpression of MIA3 led to a decrease in the apoptosis rate (Fig. 3I). We showed that overexpression of MIA3 inhibited the expression of E-cadherin and cleaved caspase-3 in Huh7 cells. Increased expression of N-cadherin, Vimentin, and CyclinD was observed (Fig. 3K).

### MIA3 knockout inhibits the proliferation and migration of HCC cells

**Fig. 4:**
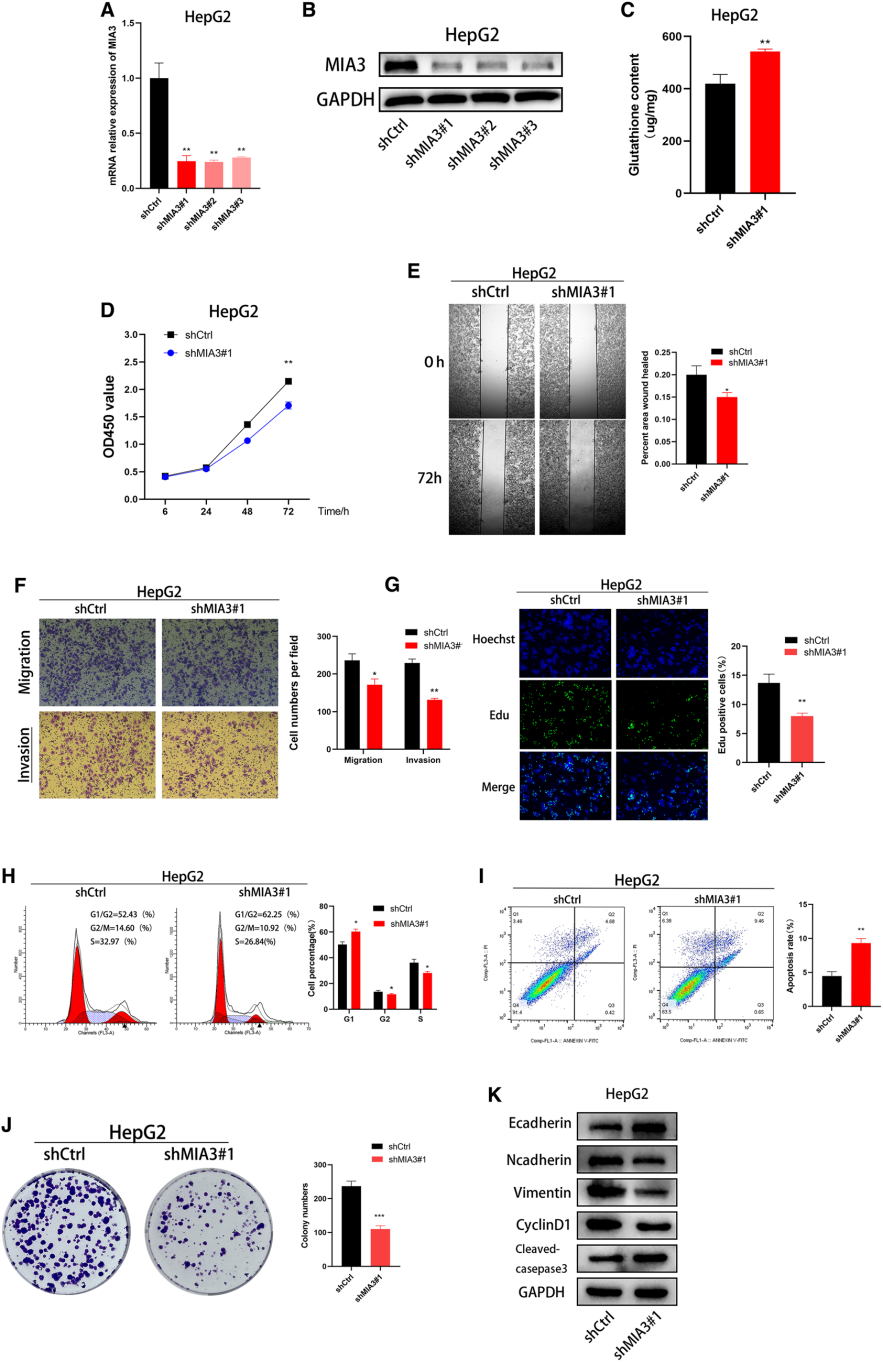
Knocking out MIA3 inhibits HCC cell growth. **A**, **B** After MIA3 lentivirus transfection of Hep-G2 cells, MIA3 mRNA and protein expression was measured by qRT-PCR and Western blot experiments. **C** Detection of glutathione expression in HCC cells after knockout of MIA3. **D** CCK-8 experiments to detect the proliferation function of HCC cells after knocking out MIA3. **E** Detection of the migration capacity of HCC cells with MIA3 knockout by wound healing assay. **F** Detection of the migration and invasion ability of HCC cells with MIA3 knockout by the transportation method. **G** EdU experiments to detect the proliferation function of HCC cells after knocking out MIA3. **H**, **I** Flow cytometry analysis of the cell cycle distribution and apoptosis ratio of Hep-G2 cells. **J** Colony formation experiment to detect the proliferation function of HCC cells after knocking out MIA3. **K** Western blotting was used to detect proteins related to apoptosis, the cell cycle, and EMT in Hep-G2 cells. An unpaired two-tailed Student’s *t* test was used. All data are shown are from 3 independent experiments; (*p < 0.05, **p < 0.01, ****p < 0.001)

Based on the expression level of MIA3 in HCC cell lines, we established a stable model of MIA3 knockout in Hep-G2 cells by lentiviral transduction. qRT-PCR and Western blot results showed that MIA3 was effectively knocked out in Hep-G2 cells (Fig. 4A, B). In Hep-G2 cells with MIA3 knockout, the expression level of glutathione in the MIA3 knockout group was higher than that in the control group of MIA3 (Fig. 4C). CCK-8 colony formation and EdU experiments showed that knocking out MIA3 inhibited the proliferation of Hep-G2 cells (Fig. 4D, G, J). Cell migration and invasion experiments showed that knocking out MIA3 inhibited the migration and aggressiveness of HCC cells (Fig. 4E, F). The cell cycle distribution also showed that knocking out MIA3 increased the number of cells in the G1/G0 phase and decreased the number of cells in the G2/M and S phases (Fig. 4H). Apoptosis experiments showed that knocking out MIA3 led to an increase in the apoptosis rate (Fig. 4I). We showed through Western blot experiments that knocking out MIA3 increased the expression of E-cadherin and cleaved caspase-3 in Hep-G2 cells. Reduced expression of N-cadherin, Vimentin, and CyclinD was observed (Fig. 4K).

### Overexpression of MIA3 enhances the tumorigenicity of HCC cells in vivo

**Fig. 5:**
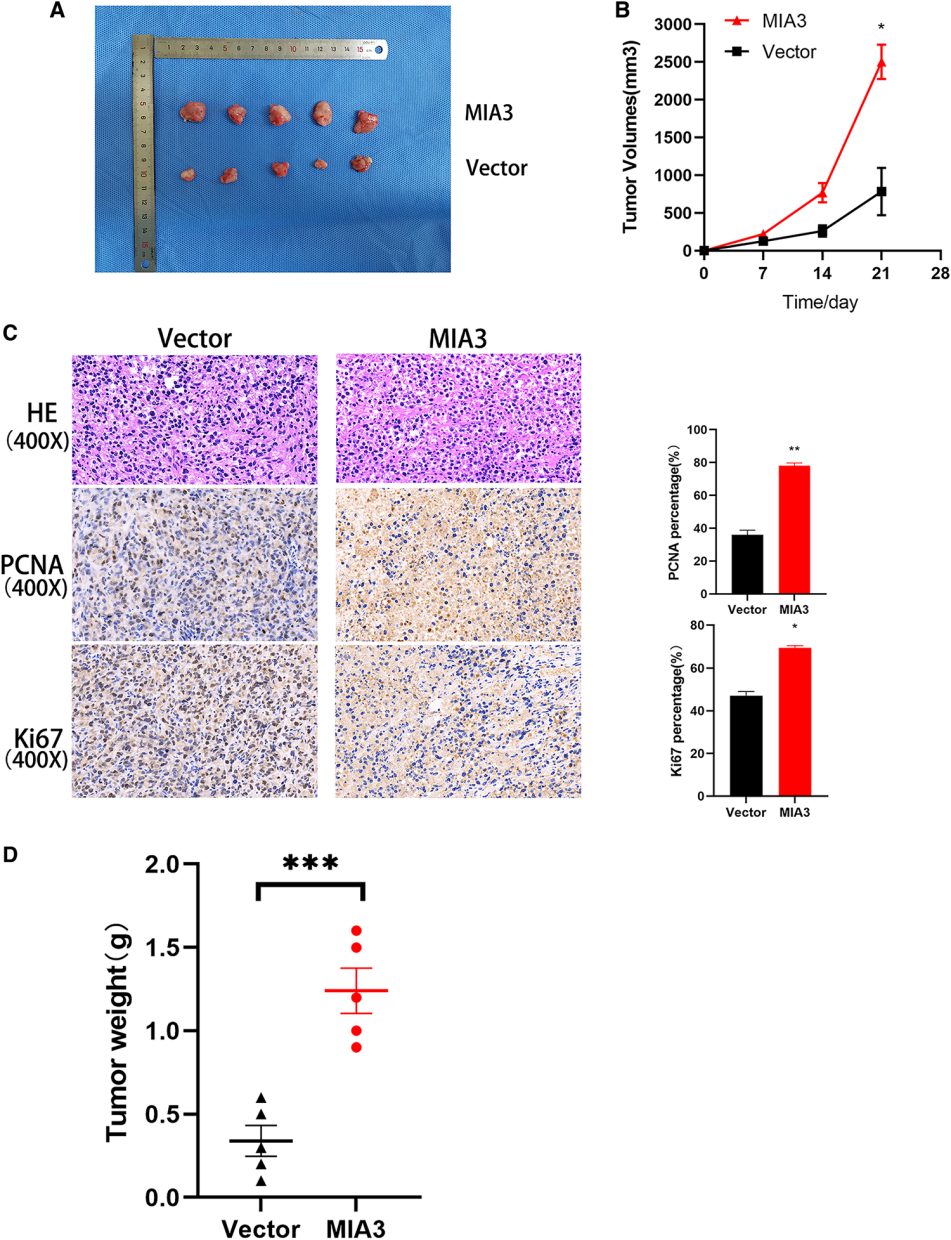
MIA3 promotes the proliferation of HCC cells in vivo. **A** Tumour image after subcutaneous injection of 1 × 10^7^ cells in nude mice MIA3-Huh7 cells or control group Huh7 cells. **B** Data for each group of tumour nodules were measured weekly. **C** IHC detected representative HE staining and expression of Ki67 and PCNA. **D** The average tumour weight of the MIA3 group increased compared with that of the control group. An unpaired two-tailed Student’s *t* test was used. All data are expressed as the mean ± standard deviation. (*p < 0.05, **p < 0.01, ****p < 0.001)

We further explored the effect of MIA3 on cell proliferation in vivo, for which we established a subcutaneous xenograft model. MIA3-overexpressing Huh7 cells (1 × 10^7^) were mixed with PBS and injected into 3- to 4-week-old female BALB/c nude mice. Its effect on tumour growth was assessed by measuring tumour size after injection. Overexpression of MIA3 in Huh7 cells significantly promoted cell proliferation (Fig. 5A, B, D). In addition, we detected the expression of Ki67 and PCNA proliferation markers at the tissue level. The IHC results showed that the expression of Ki67 and PCNA in the MIA3 overexpression group was higher than that in the control group (Fig. 5C). This result confirms that MIA3 promotes HCC cell-induced tumour growth and that MIA3 plays a key role in HCC cell proliferation in vivo.

### MIA3 further promotes the degradation of glutathione by binding to CHAC1

**Fig. 6:**
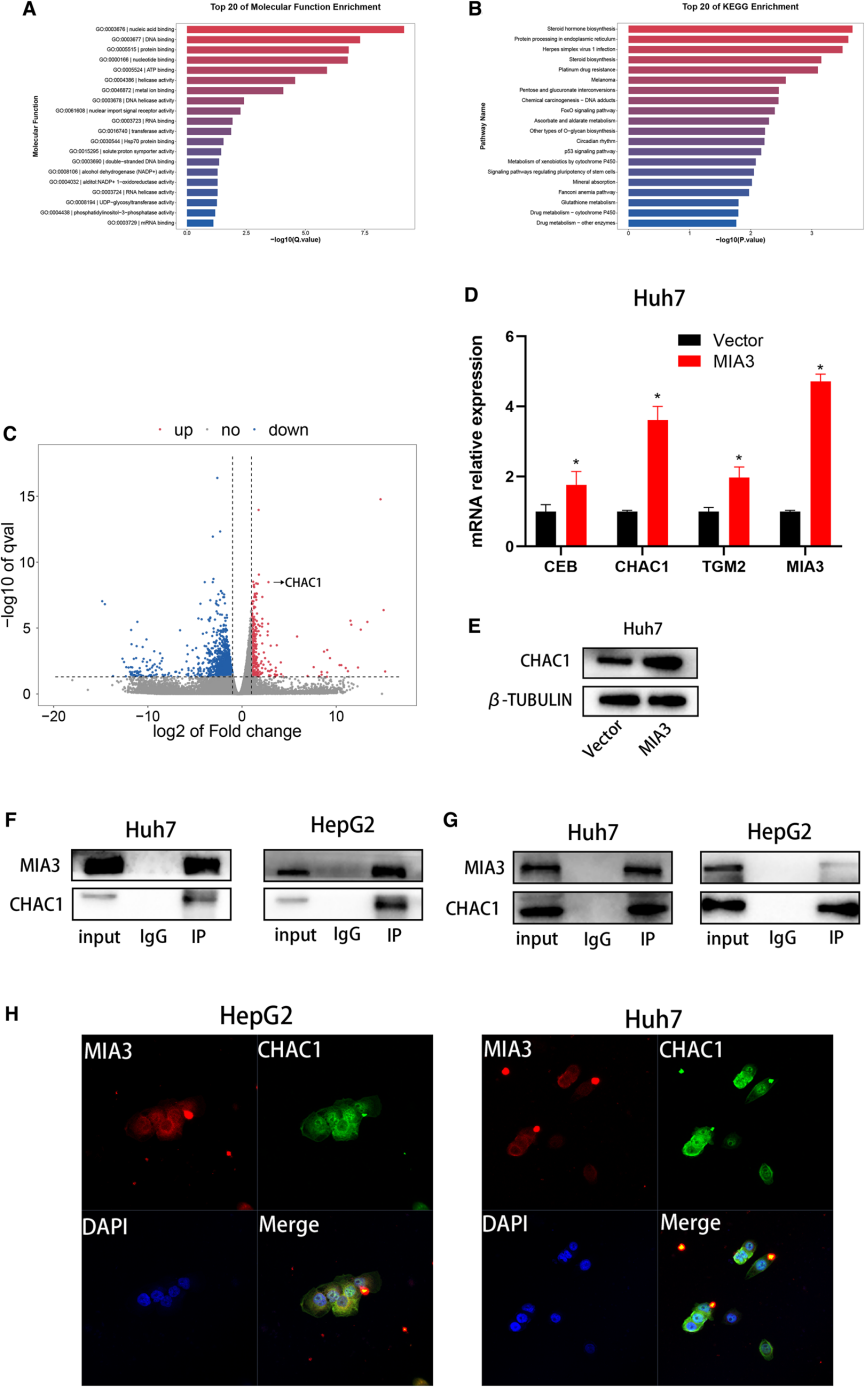
The MIA3 and CHAC1 proteins interact. **A** GO function enrichment map for transcriptomics. **B** KEGG pathway enrichment map for transcriptomics. **C** Volcano plot of transcript enrichment. **D** The qRT-PCR results showed that the expression of CHAC1 was upregulated in Huh7 cells with MIA3 overexpression. **E** The results of Western blot experiments showed that the expression of CHAC1 was upregulated in Huh7 cells with MIA3 overexpression. **F** COIP experiments showed that MIA3 and CHAC1 had protein interactions. **G** COIP and pull down experiments showed that the MIA3 and CHAC1 proteins interacted. **H** Confocal microscopy images showing the colocalization of MIA3 and CHAC1 in the cytoplasm of Huh7 and Hep-G2 cells

To further investigate the potential molecular mechanisms of MIA3 in HCC cells, we constructed Hep-G2 cells overexpressing MIA3 or a control vector for RNA-seq. The RNA-seq results revealed 1789 differentially expressed genes, including 314 upregulated genes and 1475 downregulated genes. GO enrichment analysis showed the function of the first 20 molecules, and the data showed that genes upregulated in MIA3-overexpressing cells were enriched in the protein binding process (Fig. 6A). KEGG enrichment analysis showed the top 20 pathways that were enriched. The data show that upregulation of MIA3 induces activation of protein processing-related pathways in the endoplasmic reticulum (Fig. 6B). The volcano plot shows the top 20 upregulated genes and downregulated genes (Fig. 6C). Among the upregulated genes, CHAC1, CEBPB, and TGM2 were significantly upregulated, and we further verified these results in Huh7 cells with qRT-PCR. The verification showed that in Huh7 cells overexpressing MIA3, CHAC1, CEBPB, and TGM2 expression was upregulated (Fig. 6D). To further investigate the potential relationship between MIA3 and CHAC1, we performed localization analysis, and both proteins were localized in the endoplasmic reticulum. Next, coimmunoprecipitation (Co-IP) experiments and confocal fluorescence microscopy experiments were performed, and the experimental results showed that there was a protein interaction between MIA3 and CHAC1 (Fig. 6F–H). The results of Western blot experiments showed that CHAC1 expression was upregulated in Huh7 cells overexpressing MIA3 (Fig. 6E).

### Knockout of CHAC1 inhibits the proliferation and migration of HCC cells overexpressing MIA3

**Fig. 7:**
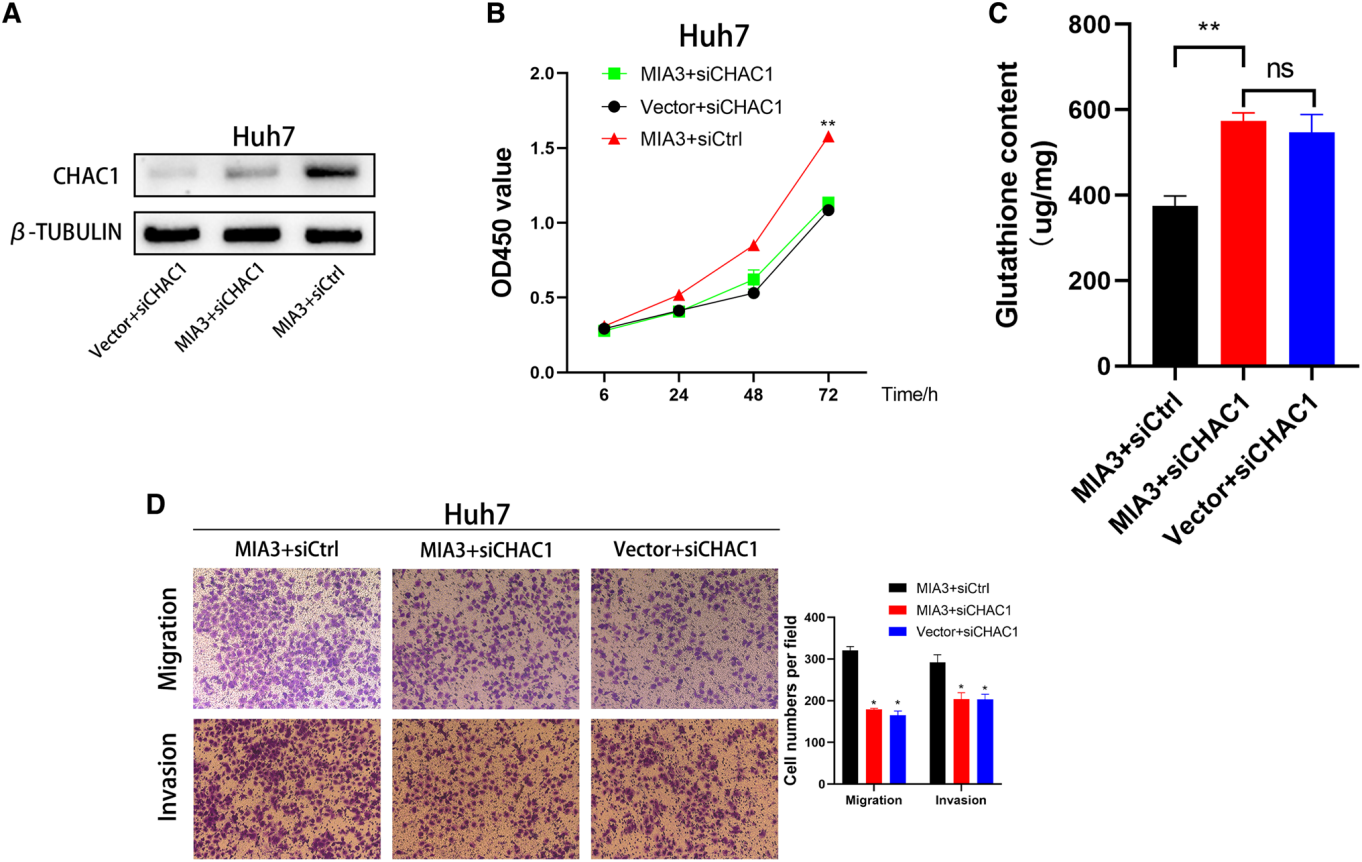
CHAC1 knockout inhibits MIA3-mediated HCC proliferation and migration invasion. **A** Western blot detection of protein expression in HCC cells overexpressing MIA3 after transfection with vector, siCtrl, or siCHAC1. **B** CCK-8 experiments showed that downregulation of CHAC1 could inhibit the proliferation of HCC cells overexpressing MIA3. **C** Detection of glutathione content in HCC cells overexpressing MIA3 and transfected with control vectors siCtrl or siCHAC1. **D** Effects of MIA3-expressing HCC cells and vector-transfected siCtrl or siCHAC1 on migration and invasion. Unpaired, two-tailed students *T* test. All data are shown are from 3 independent experiments (*p < 0.05, **p < 0.01, ****p < 0.001.)

MIA3 further promotes the proliferation, migration, and invasion of HCC cells by binding to CHAC1 to promote the degradation of glutathione (GSH). We knocked down CHAC1 in HCC cells that overexpressed MIA3. The Western blotting results showed a significant decrease in the expression of MIA3 (Fig. 7A), and knockdown of CHAC1 increased the expression of glutathione in Huh7 cells overexpressing MIA3 (Fig. 7C). Importantly, CCK-8, cell migration and invasion experiments analysis showed that knockdown of CHAC1 could inhibit the proliferation, migration and aggressiveness of Huh7 cells overexpressing MIA3 (Fig. 7B, D).

## Discussion

Hepatocellular carcinoma is the main type of liver cancer. The insidious onset of liver cancer, lack of effective treatment, and poor prognosis have attracted widespread attention [20]. Therefore, in-depth investigation of the pathogenesis of HCC will help to further improve the treatment of HCC, improve survival prognosis, and prolong the survival time of patients. MIA3 is a member of the MIA gene family, which also includes MIA, MIA2, and OTOR, which have significant homology and similar gene organization at the nucleotide and protein levels; they have 34–45% amino acid identity and 47–59% cDNA sequence identity and share a highly conserved SH3 domain [21]. The MIA gene family is closely related to tumours. According to the latest studies, MIA is associated with the progression, metastasis, and poor prognosis of malignant tumours such as melanoma, pancreatic cancer, and gastric cancer [22–25]. MIA2 is expressed at low levels in HCC and controlled by the hepatocyte nuclear factor (HNF)1 binding site in the MIA2 promoter [26]. OTOR promotes tumour growth in breast cancer by regulating mitogen-activated protein kinase/extracellular signal-regulating kinase-extracellular signal-regulating kinase (MEK-ERK) signalling [27]. In recent studies, MIA3 was shown to promote tumorigenesis. MIA3 is an inhibitor in malignant melanoma [28] and colorectal cancer [4, 5] and is a promoter of tumour growth in oral squamous cell carcinoma [3]. MIA3 is expressed in tumour tissues of prostate cancer patients, has high sensitivity and specificity; it can be used as a new diagnostic biomarker for prostate cancer [29]. TCGA database analysis revealed high expression of MIA3 in liver cancer tissues. Our experimental study found that tumour tissue cells had higher MIA3 expression than normal liver cells. In summary, the MIA3 expression level is positively correlated with the occurrence of HCC, indicating that MIA3 is a tumour promoter in HCC.

CHAC1 is a member of the γ-glutamyl cyclotransferase family of proteins, and CHAC1 catalyses the cleavage of glutathione into 5-oxo proline and the dipeptide cysteinylglycine. It has a specific effect on glutathione but not other γ-glutamyl peptides [30]. GSH depletion is an important factor in apoptosis initiation and execution. CHAC1 acts as a proapoptotic component of the unfolded protein response pathway by mediating the proapoptotic effect of the ATF4-ATF3-DDIT3/CHOP cascade [12]. CHAC1 is also involved in the negative regulation of the Notch signalling pathway of embryonic neurogenesis by inhibiting Notch S1 furin-like cleavage to prevent Notch cell surface presentation, maintaining Notch in an immature inactive form and thereby promoting neurogenesis in embryos [31]. CHAC1 inhibits cell viability and increases the sensitivity of prostate cancer cells to DTX by inducing ER stress and iron death in prostate cancer [32]. In liver cancer, upregulation of CHAC1 expression is also closely related to disease progression and poor prognosis. [33–35]

In this study, we found that the expression of the MIA3 gene in the tissues of liver cancer patients was increased through the mining of TCGA data, and immune infiltration analysis revealed that MIA3 was negatively correlated with immune cells in HCC. The correlation between high expression of MIA3 in HCC tissues and low infiltration of immune T cells was verified by analysing immune tissue chips. We also observed that upregulating MIA3 enhances HCC cell proliferation, migration, and invasion capacity, and knocking out MIA3 inhibits HCC cell proliferation, migration, and invasion capacity. To further explore the potential mechanism of MIA3 in HCC cells, we constructed overexpressed HCC cells for RNA-seq experiments, and the analysis of RNA-seq results showed that compared with HCC cells in the control group, the molecular functions and signalling pathways of cells overexpressing MIA3 were significantly activated in protein binding and endoplasmic reticulum protein processing. CHAC1 was among the top 20 upregulated genes shown in the volcano map, and the CHAC1 and MIA3 genes are both localized to the endoplasmic reticulum in cells. To verify their interaction, we performed Co-IP and confocal fluorescence microscopy experiments, and the experimental results showed that the MIA3 and CHAC1 proteins interacted. We also observed that in HCC cells overexpressing MIA3, knocking out the CHAC1 gene inhibited glutathione degradation, increased glutathione expression, and decreased the ability of HCC cells to proliferate, migrate, and invade.

Taken together, these study data suggest that MIA3 expression is upregulated in HCC tissues. Overexpression of MIA3 can promote HCC cell proliferation, migration, and invasion and HCC tumorigenesis. MIA3 exerts tumour-promoting effects in HCC by binding to the CHAC1 protein and promoting glutathione biodegradation. In terms of mechanism, overexpression of MIA3 increases CHAC1 expression and promotes glutathione degradation in HCC to further promote the occurrence and development of liver cancer. Therefore, we believe that MIA3 is a valuable potential therapeutic target for HCC.

## Conclusion

In summary, MIA3 is an oncogene that promotes the occurrence and development of HCC by interacting with the CHAC1 protein and promoting the degradation of glutathione. MIA3 may become a new target for the diagnosis and treatment of hepatocellular carcinoma.

## Data Availability

The datasets provided in this study can be found in the online repository. The data described herein are included in published articles, or experimental data may be provided upon reasonable request.

## Abbreviations

MIA3: Melanoma inhibition active protein 3
TCGA: Cancer genome atlas
HCC: Hepatocellular carcinoma
CHAC1: Glutathione-specific γ-glutamyl cyclotransferase 1
qRT-PCR: Real-time quantitative reverse transcription polymerase chain reaction
CCK-8: Cell Counting Kit-8
Co-IP: Coimmunoprecipitation
shRNA: Short hairpin RNA
